# Role of Health Literacy in Self-Reported Musculoskeletal Disorders

**DOI:** 10.1155/2015/607472

**Published:** 2015-08-18

**Authors:** Catherine L. Hill, Sarah L. Appleton, Julie Black, Elizabeth Hoon, Rima E. Rudd, Robert J. Adams, Tiffany Gill

**Affiliations:** ^1^Rheumatology Unit, The Queen Elizabeth Hospital, 28 Woodville Road, Woodville South, SA 5011, Australia; ^2^University of Adelaide, Adelaide, SA 5005, Australia; ^3^Arthritis South Australia, 118 Richmond Road, Marleston, SA 5033, Australia; ^4^Department of Social and Behavioral Sciences, Harvard School of Public Health, 677 Huntington Avenue, Kresge Building 7th Floor, Boston, MA 02115, USA; ^5^Population Research & Outcomes Unit, University of Adelaide, Adelaide, SA 5005, Australia

## Abstract

Self-report of musculoskeletal conditions is often used to estimate population prevalence and to determine disease burden and influence policy. However, self-report of certain musculoskeletal conditions is frequently inaccurate, suggesting inadequate communication to the patient of their diagnosis. The aim of this study is to determine the association between functional health literacy (FHL) and self-reported musculoskeletal conditions in a representative population survey. FHL was measured using Newest Vital Sign in 2824 randomly selected adults. Participants also self-reported medically diagnosed arthritis, gout, and osteoporosis. Multiple logistic regression was adjusted for age and sex. The prevalence of self-reported arthritis, gout, and osteoporosis was 25.2%, 4.9%, and 5.6%, respectively. The prevalence of those at risk for inadequate FHL was 24.0% and high likelihood of inadequate FHL was 21.0%. However, over 50% of respondents with arthritis or gout had at risk/inadequate FHL, increasing to 70% of those self-reporting osteoporosis. After adjustment for age and sex, respondents in the arthritis subgroup of “don't know” and self-reported osteoporosis were significantly more likely to have inadequate FHL than the general population. This study indicates a substantial burden of low health literacy amongst people with musculoskeletal disease. This has implications for provider-patient communication, individual healthcare, population estimates of musculoskeletal disease, and impact of public health messages.

## 1. Background

Chronic disease management requires that patients regularly undertake multiple tasks that involve sophisticated literacy skills, speaking and listening skills in the clinical encounter, numeracy skills for calibrating medicine dosage and timing, and reading skills for understandings issues, directions, and plans. Management of arthritis offers an additional challenge because of different arthritis subtypes, gout, or osteoporosis. Correct knowledge of disease has important implications in terms of self-management of these disorders. The plethora of information now more widely available to the public in print and online makes correct knowledge of diagnosis and disease entity critically important.

Links between literacy skills of patients and health outcomes, especially related to chronic disease, are now well established [1]. Functional health literacy (FHL) involves the ability to read, calculate, and act on oral and written information in healthcare settings [2]. It has been described as the “*final neglected pathway to high-quality healthcare*” [2]. Limited FHL is associated with adverse health outcomes, less frequent preventive health behaviors, less active self-management of chronic conditions, premature mortality, and higher healthcare costs [3, 4]. Population studies from developed countries, including the USA and Australia, have demonstrated that approximately 50% of adults have limited FHL skills, achieving a score below that needed to use health related materials found in everyday life with accuracy and consistency [5–7].

Arthritis covers a wide range of musculoskeletal conditions, which is somewhat unique among chronic diseases. Self-care and management rely on the patient's knowledge of his or her specific diagnosis. Previous work has demonstrated that over 30% of those having arthritis are unaware of the type of arthritis they have and that this is related to low socioeconomic indicators such as low income and poorer educational status [8]. In addition, previous studies have demonstrated that the accuracy of self-report varies between musculoskeletal conditions. For example, a recent study of self-reported medically diagnosed RA found that this was only accurate in 14.7% of cases and is also frequently inaccurate in people who self-report medically diagnosed osteoporosis [9–11]. In contrast, recent work using 2 large community cohorts demonstrated that self-report of physician-diagnosed gout had good reliability and sensitivity [12]. An understanding of factors contributing to this inaccuracy can help shape communication strategies in the clinical encounter and for more general public health efforts. Thus, the aim of this study was to determine the association of functional health literacy and self-report of 3 common musculoskeletal conditions (arthritis and its subtypes, gout, and osteoporosis) in a population-based survey.

## 2. Methods

Data was obtained from the South Australian Health Omnibus Survey (SAHOS) during spring 2008. Within each Australian Bureau of Statistics collector district a random starting point was selected and ten households were sampled using a fixed skip interval. In a nonreplacement sample one adult aged fifteen years or older, whose birthday was next, was selected for interview in their home by trained health interviewers. The SAHOS methodology has been described in detail elsewhere [13]. The 2008 population sample was 2824 from 4614 households contacted (61.2% participation rate) and the sociodemographic distribution of participants corresponded to SA population estimates.

Respondents completed the Newest Vital Sign as a measure of FHL. The NVS [14] is a screening test developed specifically for use in primary care. It consists of a nutrition label for ice cream, accompanied by 6 questions assessing the participant's ability to use the information on the label to make decisions and determinations. Compared with the most commonly used HL instrument, the Test of Functional Health Literacy in Adults (TOFHLA) [15], the NVS has a high sensitivity for detecting limited health literacy [15] beyond that of education and age alone [14]. Compared to the TOFHLA, the specificity of the NVS may produce overestimates of limited FHL [14]. The NVS is scored out of 6, with a score of 4–6 almost always indicating adequate health literacy (described as “adequate” in this paper), a score of 2-3 indicating the possibility of limited health literacy (“at risk”) and a score of 0-1 indicating a high likelihood (50% or more) of limited health literacy (“inadequate”) [14]. It has been used in both clinical and population settings and has acceptable responder burden with mean completion time of under 3 minutes [14, 16].

Respondents were also asked a range of health-related questions. Prevalence of self-reported arthritis, gout, and osteoporosis was determined by asking about diagnosis and type (Table 1).

Demographic data included gender, education level, employment status, household income, country of birth, and area of residence.

The questionnaire and methodology for this survey were approved by the Committee for the Ethics of Human Research of the North Western Adelaide Health Service (The Queen Elizabeth Hospital) and the South Australian Department of Health Ethics Committee. Each participant gave written informed consent.

### 2.1. Statistical Analysis

Data was analyzed using the Statistic Package for the Social Sciences (SPSS Version 15.0, SPSS Inc., Chicago, IL) and weighted to the individual's probability of selection and to Australian Bureau of Statistics population estimates [17]. Bivariate associations of the 3 conditions with demographics and health literacy were determined with Chi-square tests. Multiple logistic regression models were developed for outcome variables of inadequate health literacy (NVS scores 0-1) compared with those with adequate health literacy (scores 4–6) and also for adequate health literacy compared to those either at risk or with inadequate (0–3) health literacy.

## 3. Results

Of the 2824 participants, 1358 (48.1%) were male and 2158 (76.4%) resided in the metropolitan area of Adelaide. Participants who did not respond to an individual relevant questions were excluded from the analysis (arthritis question *n* = 9, gout question *n* = 16, and osteoporosis *n* = 17). Of those who reported arthritis, 40.3% reported having OA, 18.9% RA, 36.1% “don't know,” and 4.7% “other.” Presence of self-reported medically diagnosed arthritis (25.2%), gout (4.9%), and osteoporosis (5.6%) was associated with increasing age and markers of social disadvantage (Table 2). In the whole sample, the prevalence of those at risk for limited FHL was 24.0% and of a high likelihood of inadequate FHL was 21.0%. Over 50% of respondents with arthritis or gout had at risk or inadequate FHL which increased to 70% of those diagnosed with osteoporosis. Among those diagnosed with arthritis, those who did not know which subtype of arthritis they had were more likely to have low FHL than people in the other subgroups of arthritis (Table 2). After adjustment for age and sex, respondents in the subgroup of “don't know” and self-reported osteoporosis were significantly more likely to have low FHL than the general population (Table 3). Those in the other subcategories of arthritis were no more at risk of low FHL than the general population (Table 3), nor were those with self-reported gout.

## 4. Discussion

This study indicates that those with self-reported RA, “don't know” arthritis response, or self-reported osteoporosis were more likely to have low FHL than other subtypes of arthritis or gout. This is consistent with the findings that these are the most likely groups to have inaccurate self-report of their diagnoses. Furthermore, this study demonstrates that there is a substantial burden of low FHL amongst people with self-reported medically diagnosed arthritis, gout, and osteoporosis in the general population. Although this level of FHL is partially explained by the older age of people with these conditions, it does not detract from the significant burden that this carries for the community. One in two people in the community with arthritis and gout and two in three people with osteoporosis have low FHL. Although this study is limited by its cross-sectional nature and inability to verify musculoskeletal diagnoses, it is strengthened by the study methodology of random population sampling and face-to-face interviews.

Arthritis, gout, and osteoporosis each require significant self-management and the application of a variety of literacy skills. In addition, appropriate knowledge of the correct “arthritis” diagnosis is essential for the appropriate overall management of symptoms and disease. For example, the optimal management of both OA and RA requires a combination of medication, allied health services, and lifestyle changes. However, the treatment approaches and medication regimens in each of these subtypes are quite different. The optimal treatment of gout and osteoporosis requires accurate diagnosis and management, involving medication, diet, and lifestyle interventions. Patients mistaking their diagnosis for another diagnosis that falls under the commonly used term “arthritis” may well follow inappropriate guidelines, drawn from websites or learned from others. People with chronic musculoskeletal disorders are increasingly using online communities to help manage their conditions [18].

In addition, patients in care not only need to “take in” the information given to them by their clinicians regarding the diagnosis and management of their illness at each consultation, but also need to be able to “navigate” the health system to obtain required appointments, manage a number of healthcare providers and referrals, and comprehend complex medication regimens. Consequently, the mismatch between health literacy skills and the “sophisticated literacy skills” needed for disease management needs to be addressed [19].

Accurate communication of diagnosis by healthcare providers is an essential foundation for self-management behaviors [18]. First, clinicians must become aware of the mismatch between patient's abilities and the demand inherent in the information and the tasks asked of them. Population statistics related to adult literacy skills can serve to raise awareness and need to be purposively disseminated in the health sector. Next, doctor-patient communication around the issues of diagnosis must be closely examined and alternative strategies must be explored.

A number of techniques that clinicians can use in consultations to improve communication have been shown to help reduce the potential risks associated with limited health literacy. These strategies include avoiding the use of medical jargon, showing interest in questions, explaining forms, slowing and confirming understanding through techniques such as teach-back, and making use of visual aids [20, 21]. Several intervention programs have been widely disseminated. For example, the US-based National Patient Safety Foundation has designed the “Ask Me 3” program to promote communication between healthcare providers and patients using the 3 questions “What is my main problem? What do I need to do? Why is it important for me to do this?” [22]. Furthermore, the use of “teach-back” has been documented to improve care in diabetes [23], although this has not been tested in musculoskeletal conditions.

Programs that specifically tailor education to people with limited health literacy have been shown to improve outcomes in a range of chronic conditions such as diabetes [24–26]. A variety of strategies were used in a number of different combinations across different health conditions, including care management, simplifying language in written materials, use of pictorial information, videos, and audiotapes, specifically checking for understanding, spacing information, and training professionals in communication techniques [26].

However, the evidence for some of these strategies in musculoskeletal diseases is limited, as highlighted by a recent systematic review which found a lack of high-quality evidence on the effectiveness of musculoskeletal education interventions for people with lower literacy levels [27]. A recent randomized controlled trial of an intervention to reduce low literacy barriers in inflammatory arthritis management showed no benefit on outcomes [28]. However, the intervention may have been hindered by long disease duration and high literacy levels of those who participated [28]. In contrast, a telephone-based OA self-management support intervention was found to be effective in improving pain in those with lower health literacy [29].

An alternative approach to targeting specific information to those with low FHL which has been recommended is to adopt the principle of “universal precautions,” analogous to that used in infectious disease exposures [30]. In this model clinicians and health systems take specific actions to minimize the chances that patients may not understand all information provided by assuming everyone may have difficulties and that systems exist to promote understanding for all patients [30]. An adaptation of this type of “health literacy universal precaution toolkit” for rheumatology has recently been published for use by pharmacy professionals, although not yet tested in community pharmacy sites [31].

Self-report of musculoskeletal conditions is often used to provide population prevalence estimates and to determine disease burden and influence policy. Our findings suggest that health literacy is influencing the population estimates of musculoskeletal diseases. Policy makers need to take this knowledge into account when assigning resources to different musculoskeletal disorders. Public health practitioners developing health communication messages must be equally attentive to adult literacy skills. Individuals with low health literacy are known to have more difficulty with preventive tasks, such as screening programs [32]. This has implications for effective prevention and detection of musculoskeletal disorders such as osteoarthritis and osteoporosis which requires education regarding exercise, diet, and bone density screening. In addition, as patient reported outcome measures (PROMs) become increasingly used in rheumatological practice, health literacy needs to be taken into account when developing these tools. A recent study of 10 PROMs used in rheumatology demonstrated that only 60% met the recommended reading level for health education literature [33]. Therefore, it is likely that these PROMs will be difficult to complete for those with low FHL. However, the effect of health literacy on PROM measurements in rheumatology is not known.

## 5. Conclusion

Many patients with self-reported medically diagnosed arthritis, gout, or osteoporosis have inadequate health literacy. This is most profound among those who self-report RA or do not know the type of arthritis they have and among those with self-reported osteoporosis. These findings indicate a need for improved doctor-patient communication, not only for disease management discussions but also at the critical moment of initial diagnosis. In order to improve patient outcomes in these diseases, we must recalibrate the norm and recognize the documented limited literacy skills of a majority of adults. Furthermore, we must take into account the burden of low health literacy amongst patients with arthritis, gout, and osteoporosis, and its effects on population estimates of these disorders. Whilst we must certainly support improvements in the educational sector, those of us in the health sector cannot bring about needed changes in literacy skills; therefore, we can however develop and test new communication strategies to mitigate the effects of literacy on health outcomes.

## Figures and Tables

**Table 1:** Details of questions asked regarding self-reported musculoskeletal conditions.

Question	Response options
Have you ever been told by a doctor that you have arthritis?	(i) Yes, osteoarthritis
	(ii) Yes, rheumatoid arthritis
	(iii) Yes, other (specify)
	(iv) No, don't have arthritis
	(v) Yes, don't know type

Have you ever been told by a doctor that you have gout?	(i) Yes
	(ii) No
	(iii) Don't know

Have you ever been told by a doctor that you have osteoporosis?	(i) Yes
	(ii) No
	(iii) Don't know

**Table 2:** Prevalence [% (*n*)] of arthritis (all and by type), gout, and osteoporosis in relation to demographic factors and health literacy in *n* = 2815 participants.

	All arthritis (*n* = 709)	Arthritis type				Gout (*n* = 137)	Osteoporosis (*n* = 159)
		Osteoarthritis (*n* = 286)	Rheumatoid (*n* = 134)	“I don't know what type” (*n* = 256)	Other (*n* = 33)		
Sex							
Male	40.3 (286)	36.4 (104)	47.8 (64)	41.0 (105)	37.5 (12)	70.1 (96)	25.1 (40)
Female	59.7 (423)	63.6 (182)	52.2 (70)	59.0 (151)	62.5 (20)	29.9 (41)	74.9 (119)
Age							
≤59	44.8 (318)	38.1 (109)	58.9 (79)	42.6 (109)	63.6 (21)	34.3 (47)	27.7 (44)
≥60	55.2 (392)	61.9 (177)	41.1 (55)	57.8 (148)	36.3 (12)	65.7 (90)	72.3 (115)
Annual household income							
<$30,000	38.9 (276)	40.9 (117)	22.3 (36)	44.1 (113)	30.3 (10)	33.6 (46)	52.8 (84)
$30,000–80,000	27.8 (197)	28.0 (80)	30.6 (41)	26.5 (68)	24.2 (8)	23.3 (32)	23.3 (37)
≥$80,000	16.6 (118)	14.3 (41)	24.6 (33)	13.3 (34)	30.3 (10)	18.2 (25)	4.4 (7)
Not stated	16.8 (119)	16.8 (48)	14.9 (25)	16.4 (42)	12.1 (4)	24.8 (34)	18.2 (29)
Highest education							
Still at school	0.7 (5)	0.7 (2)	1.5 (2)	0.0 (0)	0.0 (0)	0.0 (0)	0.0 (0)
Left ≤ age 15 yrs/NS	22.1 (157)	21.3 (61)	16.4 (22)	26.9 (69)	15.1 (5)	19.7 (27)	35.2 (56)
Left > age 15 yrs	25.8 (183)	21.3 (61)	29.8 (40)	28.9 (74)	27.3 (9)	23.3 (32)	21.4 (34)
Left > age 15 yrs/still studying	3.7 (26)	1.7 (5)	8.2 (11)	3.5 (9)	3.0 (1)	0.7 (1)	1.9 (3)
Trade/diploma	39.9 (283)	44.4 (127)	36.5 (49)	35.9 (92)	42.4 (14)	40.1 (55)	30.8 (49)
Bachelor degree or higher	7.9 (56)	10.5 (30)	7.5 (10)	5.1 (13)	12.1 (4)	15.3 (21)	10.1 (16)
Health literacy							
Adequate (4–6)	43.1 (306)	49.0 (140)	41.8 (56)	35.1 (90)	57.6 (19)	40.9 (56)	29.6 (47)
At risk (2-3)	27.5 (195)	24.1 (69)	35.8 (48)	27.3 (70)	24.2 (8)	23.3 (32)	27.7 (44)
Inadequate (1-2)	29.5 (209)	26.9 (77)	22.4 (30)	37.5 (96)	18.2 (6)	35.8 (49)	42.1 (67)

**Table 3:** Unadjusted and adjusted associations of conditions with limitations in functional health literacy.

	At risk or inadequate FHL			Inadequate FHL		
	% (*n*)	Unadjusted	Adjusted^*∗*^	% (*n*)	Unadjusted	Adjusted^*∗*^
All arthritis						
No	41.0 (864)	1.00	1.00	18.1 (381)	1.00	1.00
Yes	56.9 (404)	1.90 (1.60–2.26)	1.14 (0.93–1.38)	29.4 (209)	1.89 (1.55–2.30)	1.01 (0.81–1.27)
Arthritis type						
No arthritis	41.0 (864)	1.00	1.00	18.1 (381)	1.00	1.00
Osteoarthritis	51.0 (146)	1.50 (1.17–1.93)	0.77 (0.58–1.02)	26.9 (77)	1.68 (1.26–2.23)	0.81 (0.59–1.13)
Rheumatoid	58.2 (78)	1.98 (1.39–2.82)	1.46 (1.01–2.14)	22.4 (30)	1.29 (0.84–1.96)	0.82 (0.52–1.29)
Don't know type	65.0 (167)	2.67 (2.04–3.50)	1.58 (1.18–2.13)	37.5 (96)	2.72 (2.07–3.59)	1.47 (1.08–2.00)
Other	40.6 (13)	0.98 (0.49–1.98)	0.71 (0.34–1.50)	18.2 (6)	0.92 (0.37–2.32)	0.63 (0.24–1.65)
Gout						
No	44.3 (1182)	1.00	1.00	20.3 (542)	1.00	1.00
Yes	59.1 (81)	1.80 (1.27–2.55)	0.96 (0.66–1.41)	35.5 (49)	2.16 (1.50–3.10)	1.09 (0.74–1.62)
Osteoporosis						
No	43.5 (1152)	1.00	1.00	19.5 (517)	1.00	1.00
Yes	70.3 (111)	3.05 (2.15–4.32)	1.67 (1.15–2.44)	42.1 (67)	3.00 (2.16–4.18)	1.62 (1.13–2.33)

^*∗*^Adjusted for age and sex.

## Abbreviation

FHL: Functional health literacy.
